# Differentiating Mobile Masses on Transcatheter Aortic Valve: Thrombi or Vegetations?

**DOI:** 10.1155/cric/9915565

**Published:** 2025-05-05

**Authors:** Alexander Arreguin, Akash Goyal, Konstantinos Dean Boudoulas, Adam Potter, Timothy Obarski

**Affiliations:** Department of Cardiovascular Medicine, The Ohio State University Wexner Medical Center, Columbus, Ohio, USA

**Keywords:** anticoagulation, aortic valve, bioprosthetic, endocarditis, thrombosis, transcatheter

## Abstract

A 58-year-old female presented with new-onset dyspnea. Two years prior, she had undergone a transcatheter aortic valve replacement with a 26-mm Edwards Sapien 3 valve. Diagnostic testing included transthoracic and transesophageal echocardiograms that revealed increased transvalvular gradients and suspected prosthetic thrombosis. Laboratory testing included blood cultures that unexpectedly grew *Streptococcus sanguinis*. This case highlights the difficulty in differentiating prosthetic valve thrombosis from infective endocarditis and the possible therapeutic complications that could arise.

## 1. History of Presentation

A 58-year-old female presented with new-onset of New York Heart Association functional Class III heart failure symptoms beginning 1 week prior after she was the restrained driver in a high-speed frontal motor vehicle collision. Two days prior to her motor vehicle collision, she had completed her ninth week of pulmonary rehabilitation, where she exercised over 20 min on a treadmill with a minimum oxygen saturation of 90% on room air and no symptoms. Upon admission, her temperature was 98.2°F, heart rate was 91 beats/min, blood pressure was 102/67 mm Hg, and respirations were 26 per minute with oxygen saturation of 61% on room air and 96% on 6 L/min of supplemental oxygen. Physical examination revealed a systolic murmur at the right upper sternal border and bibasilar crackles with diffuse end-expiratory wheezing. There was tenderness across the left chest wall. Tooth decay was present in her bilateral mandibular molars. Chest computed tomography (CT) demonstrated nondisplaced fractures of the left second to fourth ribs, evidence of pulmonary contusion, pulmonary edema, and small bilateral pleural effusions.

## 2. Past Medical History

**Figure 1:**
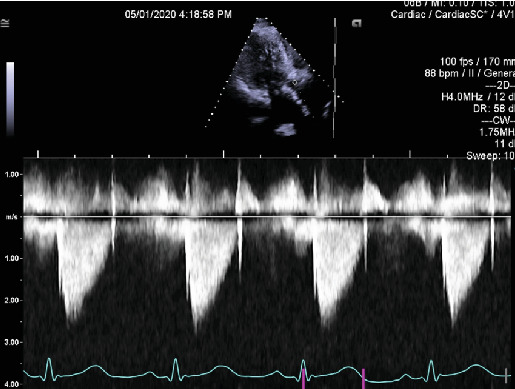
Continuous wave Doppler in the apical three-chamber view by transthoracic echocardiography. Mean transvalvular gradient of 15 mmHg and trace paravalvular regurgitation.

The patient's medical history included decompensated liver cirrhosis, chronic obstructive pulmonary disease (COPD), Stage IIIa chronic kidney disease, rheumatoid arthritis not on immunosuppression, and tobacco use (40 pack-year history). Eight months prior to her current admission, she had right facial cellulitis treated with a 10-day course of amoxicillin/clavulanic acid followed by full maxillary teeth extraction and bone shave for dentures. She underwent transfemoral transcatheter aortic valve replacement (TAVR) with a 26-mm Edwards Sapien 3 valve 2 years earlier due to symptomatic severe aortic stenosis. Periprocedural fluoroscopy demonstrated a well-positioned valve with trace regurgitation (Supporting Information 1: Video S1). Postprocedural echocardiography obtained on the day of implantation showed a mean transvalvular gradient of 15 mm Hg and trace paravalvular aortic regurgitation (Supporting Information 2: Video S2; Figure 1).

## 3. Differential Diagnosis

Life-threatening complications such as thoracic aortic injury, aortic dissection, myocardial contusion, cardiac tamponade, and aortic valve compromise were considered due to the presence of blunt chest trauma from a high-speed frontal collision. Given the patient's new clinical heart failure after the collision, the differential diagnosis included transcatheter heart valve dysfunction.

## 4. Investigations

**Figure 2:**
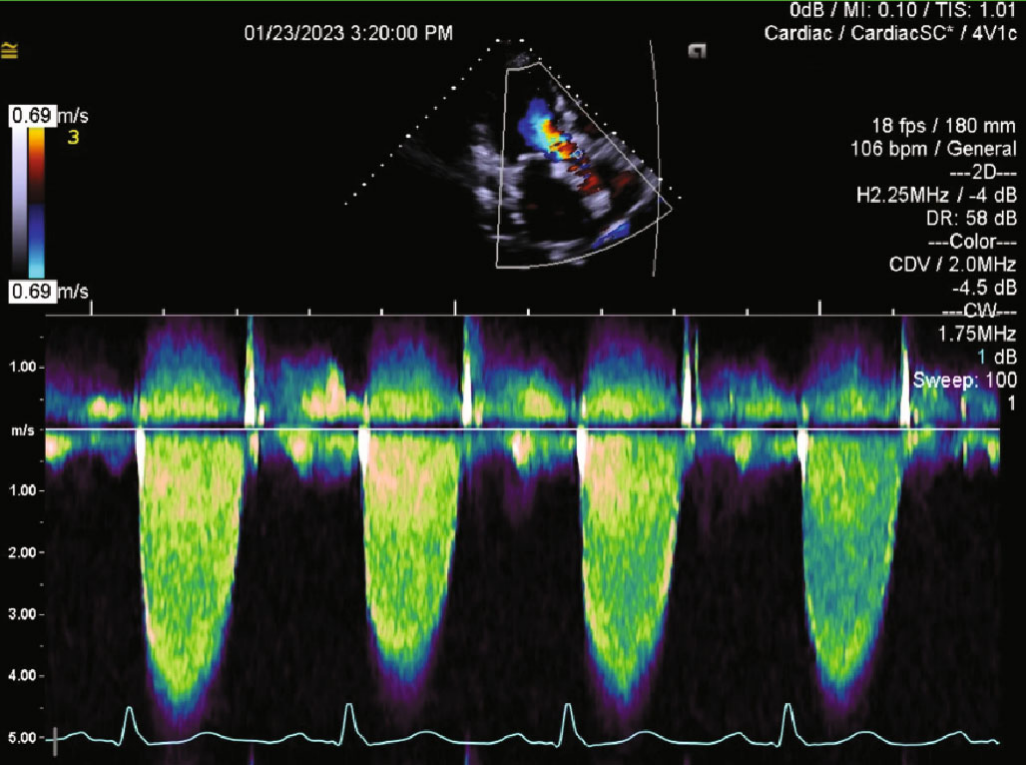
Continuous wave Doppler in the apical three-chamber view by transthoracic echocardiography. Mean transvalvular gradient of 51 mm Hg, peak prosthetic aortic jet velocity of 4.45 m/s, Doppler velocity index (DVI) of 0.20, and acceleration time of 104.25 ms.

Laboratory studies were significant for a creatinine concentration of 1.67 mg/dL. Transthoracic echocardiography (TTE) showed left ventricular ejection fraction of 55%–60%, mean transvalvular gradient of 51 mm Hg, peak prosthetic aortic jet velocity of 4.45 m/s, Doppler's velocity index (DVI) of 0.20, and acceleration time of 104.25 ms, concerning for new, severe prosthetic stenosis (Supporting Information 3: Video S3, Figure 2), as compared to TTE 8 months prior with mean gradient of 12 mm Hg and DVI of 0.47. Transesophageal echocardiography (TEE) showed numerous mobile masses of the aortic valve prosthesis involving all leaflets. With only two leaflets seen moving, the third leaflet was thought to be fixed by thrombus (Supporting Information 4: Video S4; Supporting Information 5: Video S5). Cardiac CT was not performed due to renal dysfunction.

## 5. Management

The patient was initiated on intravenous unfractionated heparin with the working diagnosis of TAVR thrombosis given the absence of infectious symptomology. Within 24 h of starting anticoagulation, she developed altered mental status, and CT of the head revealed subarachnoid hemorrhage. Systemic anticoagulation was, thus, discontinued. Two of two blood cultures subsequently grew *Streptococcus sanguinis*, suggesting subacute prosthetic IE in the setting of poor dentition.

## 6. Discussion

TAVR-IE is a rare complication, with reported incidence rates of 0.57 and 0.30 per 100 person-years for early (< 1 year) and late (> 1 year) endocarditis after TAVR, respectively [1]. Fever is the most frequent presenting symptom of TAVR-IE at admission (74.1%), followed by new-onset heart failure (39.7%) [2]. Clinical valve thrombosis after TAVR is also a rare phenomenon that may present in a similar manner. Latib et al. [3] reported prosthetic thrombosis in 0.61% of TAVR patients in a large multicenter registry, with most patients presenting with worsening dyspnea (65%) and increased transvalvular gradients (92%).

Given the present patient's new-onset dyspnea, absence of infectious symptoms or leukocytosis, increased transvalvular gradient, and visualization of mobile masses on the prosthesis, thrombosis was thought to be the most likely diagnosis. However, TAVR-IE may present with features of TAVR obstruction before overt signs and symptoms of infection, and Doppler's echocardiography in TAVR-IE can mimic TAVR thrombosis [4]. Consequently, differentiating between thrombi and vegetations using echocardiography is often inconclusive. Prior studies have shown that echocardiography has a low sensitivity in accurately identifying TAVR-IE. For example, in a multicenter registry that included 250 patients with IE after TAVR, echocardiography revealed the presence of vegetations in only 67.6% of patients [5]. Thus, advanced imaging techniques, such as cardiac CT, cardiac magnetic resonance, and metabolic imaging (radiolabeled white blood cell (WBC) SPECT/CT and/or 18F-FDG PET/CT imaging), should be considered in patients with inconclusive echocardiogram findings to establish or rule out the diagnosis of TAVR-IE [6]. The 2023 European Society of Cardiology guidelines for IE recommended the use of [18F]FDG-PET/CT and WBC single-photon emission computed tomography (SPECT)/CT in the diagnostic work-up of prosthetic valve endocarditis [7].

The pathogenesis of late prosthetic valve endocarditis is thought to resemble native valve endocarditis. Alterations in the valve surface can result in endothelial injury and enable deposition of microthrombi, to which microorganisms circulating in the blood, often from transient bacteremia from a mucosal or skin source, can bind resulting in an infection [8]. The present patient had a recent maxillary teeth extraction procedure as well as visualized tooth decay on exam, which likely allowed *Streptococcus sanguinis*, a member of the Viridans Streptococcus group and predominant microbiota in the oropharynx, to gain entrance into her bloodstream. Overall, TAVR-IE cases related to dental procedures are rare, with one observational cohort study reporting 4% of cases with a presumed odontological source of entry [2].

The patient had a clinical picture complicated by liver disease, COPD, and renal dysfunction, all of which could have increased her risk for developing prosthetic valve endocarditis. Mentias et al. [1] identified younger age at TAVR, male sex, end stage renal disease (ESRD), liver disease, and lung disease as predictors of developing IE after TAVR. In a study by Bjursten et al. [9], higher body surface area and estimated glomerular filtration rate < 30 mL/min/1.73 m^2^ were the two most significant risk factors for developing TAVR-IE. Procedure-related risk factors for IE following TAVR have also been identified and include repeat TAVR procedures (TAVR-in-TAVR), the presence of a residual moderate or severe aortic regurgitation, and increasing residual peak gradient after TAVR [1, 5, 10].

Anticoagulation is an extremely efficacious treatment regimen for TAVR thrombosis, and studies recommend starting anticoagulation as soon as valve thrombosis is suspected [3]. The patient in this case presentation developed intracranial hemorrhage, which can occur with embolization from IE; whether initiating systemic anticoagulation in this case would have changed this outcome is not clear. A retrospective analysis on a large multicenter cohort of left-sided IE patients demonstrated that anticoagulant treatment was significantly associated with cerebral hemorrhage (HR, 2.71; 95% CI, 1.54–4.76; *p* = 0.001) [11]. Given the difficulty in distinguishing TAVR-IE from TAVR thrombosis and the bleeding risk associated with anticoagulation in IE, blood cultures should be obtained before committing to anticoagulation in all patients with suspected TAVR thrombosis. However, delaying anticoagulation until culture results are known may increase the risk of thrombosis, morbidity, and mortality. Hence, management should involve a thorough assessment of both bleeding and thrombosis risks.

## 7. Follow-Up

The patient was treated with antibiotic therapy for TAVR-IE. Her case was reviewed by cardiac surgery, but given her medical comorbidities, her surgical risk was deemed to be unacceptably high. She developed further neurological compromise with suspected septic infarcts and hemorrhagic transformation on neuroimaging. Given her progressive decline with multisystem organ dysfunction, the patient's family elected to pursue palliative measures.

## 8. Conclusions

This case demonstrates the challenge of differentiating TAVR-IE from TAVR thrombosis in a patient with a newly increased transvalvular gradient and mobile masses on the valve prosthesis. The initial diagnosis based on echocardiography findings is often inconclusive, and TAVR-IE may present with no initial clinical signs of infection. Therefore, a high index of suspicion for IE in TAVR patients is required. Obtaining blood cultures in patients when the diagnosis is not clear can be helpful. This case also poses a therapeutic dilemma, as mobile masses on the prosthesis could very well represent thrombi, which should be anticoagulated, as compared to vegetations, which are not anticoagulated. Providers must leverage their clinical experience and carefully weigh both bleeding and thrombosis risks when making decisions about anticoagulation therapy prior to the receipt of final culture results.

## 9. Learning Objectives

• To understand the diagnostic challenge in differentiating IE from thrombosis in TAVR patients.
• To review patient-related and procedure-related risk factors for IE after TAVR.
• To discuss the potential therapeutic pitfalls of TAVR patients who are treated for suspected thrombosis before underlying IE has been ruled out.

## Nomenclature

COPD: chronic obstructive pulmonary disease
CT: computed tomography
DVI: Doppler velocity index
IE: infective endocarditis
LVEF: left ventricular ejection fraction
SAH: subarachnoid hemorrhage
TAVR: transcatheter aortic valve replacement
TEE: transesophageal echocardiography
TTE: transthoracic echocardiography

## Supporting Information

Supporting Information 1 Video S1: Periprocedural fluoroscopy postdeployment of 26 mm Edwards Sapien 3 valve. Valve is well seated with trace regurgitation.

Supporting Information 2 Video S2: Apical three-chamber view with color Doppler by transthoracic echocardiography on Day 0 postvalve implantation. No significant paravalvular stenosis and trace paravalvular regurgitation.

Supporting Information 3 Video S3: Apical five-chamber sweep with color Doppler and color compare by transthoracic echocardiography. Flow acceleration is seen across the prosthetic valve.

Supporting Information 4 Video S4: Midesophageal, long-axis cine by transesophageal echocardiography. Mobile masses seen on the aortic aspect of the prosthetic valve.

Supporting Information 5 Video S5: Midesophageal, long-axis cine with color Doppler by transesophageal echocardiography. Flow acceleration is seen across the prosthetic valve.
