# Supplementary material for: Differentiating Mobile Masses on Transcatheter Aortic Valve: Thrombi or Vegetations?
Source: Case Rep Cardiol. 2025 May 5;2025:9915565. doi: 10.1155/cric/9915565 (PMC12069840; doi:10.1155/cric/9915565)
Supplement: Supporting Information 1 — Video S1: Periprocedural fluoroscopy postdeployment of 26 mm Edwards Sapien 3 valve. Valve is well seated with trace regurgitation. [file 9915565.f1.pptx]

## Slide 1
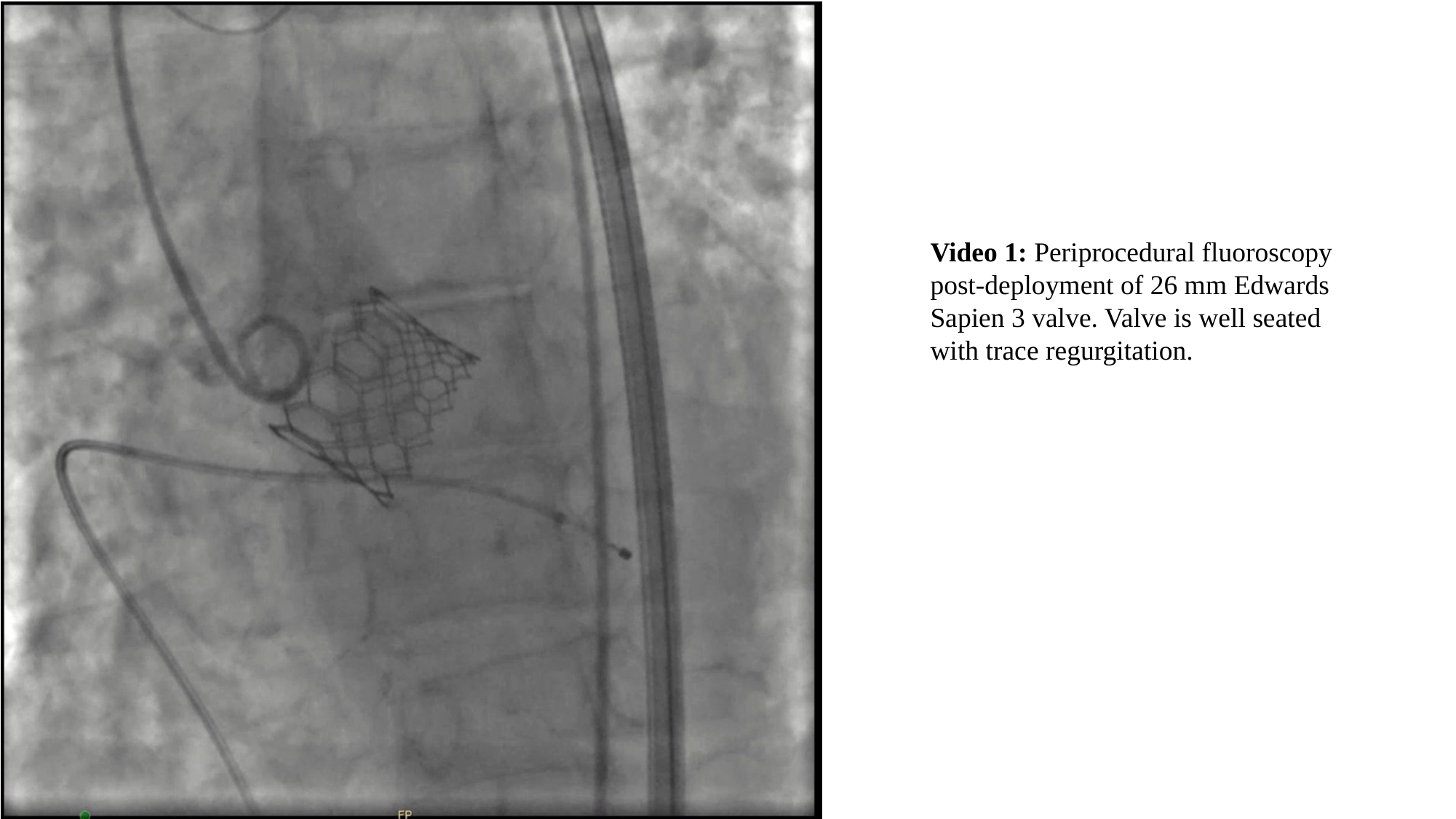

Video 1: Periprocedural fluoroscopy post-deployment of 26 mm Edwards Sapien 3 valve. Valve is well seated with trace regurgitation.
