# Supplementary material for: Differentiating Mobile Masses on Transcatheter Aortic Valve: Thrombi or Vegetations?
Source: Case Rep Cardiol. 2025 May 5;2025:9915565. doi: 10.1155/cric/9915565 (PMC12069840; doi:10.1155/cric/9915565)
Supplement: Supporting Information 2 — Video S2: Apical three-chamber view with color Doppler by transthoracic echocardiography on Day 0 postvalve implantation. No significant paravalvular stenosis and trace paravalvular regurgitation. [file 9915565.f2.pptx]

## Slide 1
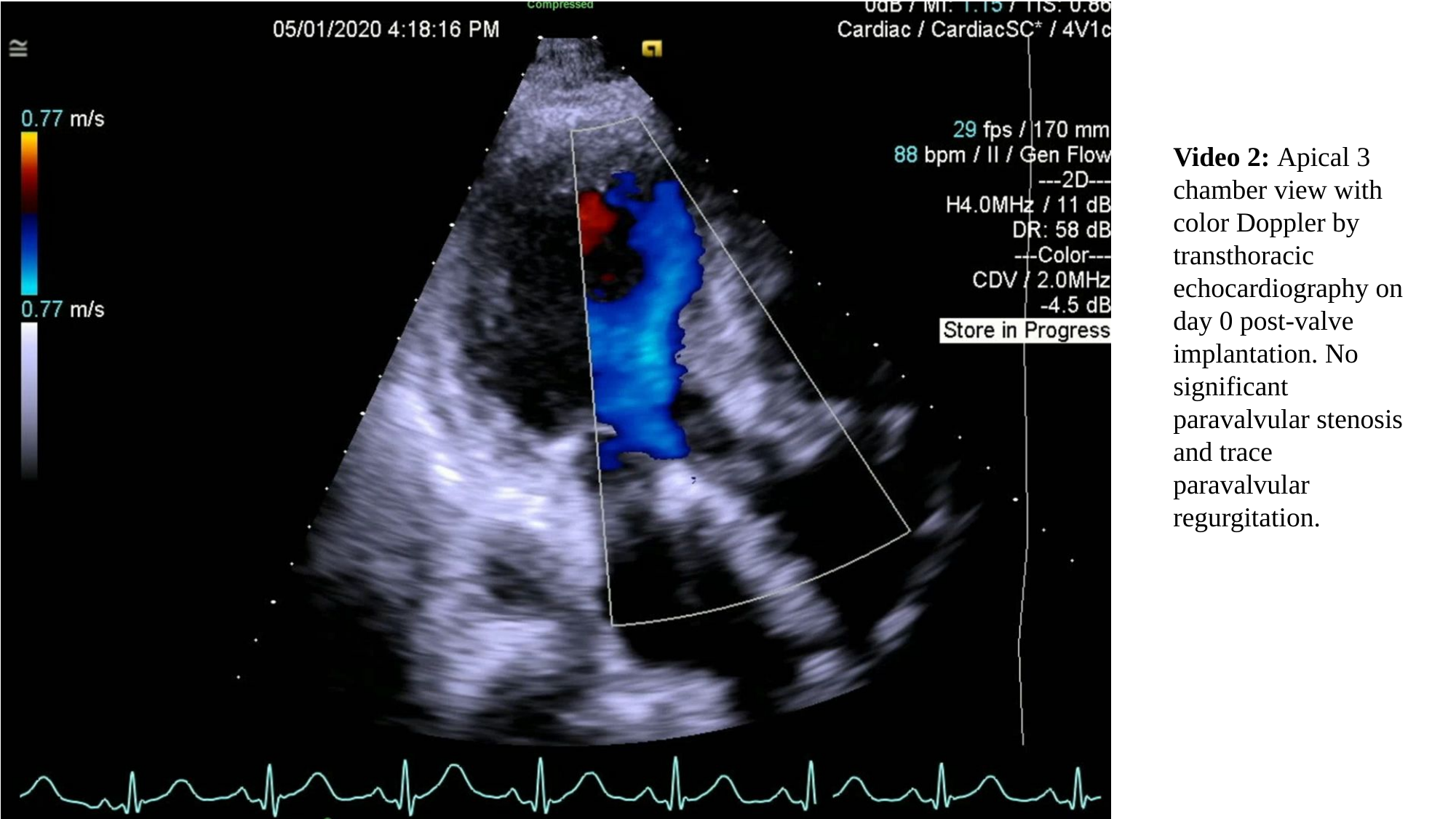

Video 2: Apical 3 chamber view with color Doppler by transthoracic echocardiography on day 0 post-valve implantation. No significant paravalvular stenosis and trace paravalvular regurgitation.
