# Supplementary material for: Differentiating Mobile Masses on Transcatheter Aortic Valve: Thrombi or Vegetations?
Source: Case Rep Cardiol. 2025 May 5;2025:9915565. doi: 10.1155/cric/9915565 (PMC12069840; doi:10.1155/cric/9915565)
Supplement: Supporting Information 3 — Video S3: Apical five-chamber sweep with color Doppler and color compare by transthoracic echocardiography. Flow acceleration is seen across the prosthetic valve. [file 9915565.f3.pptx]

## Slide 1
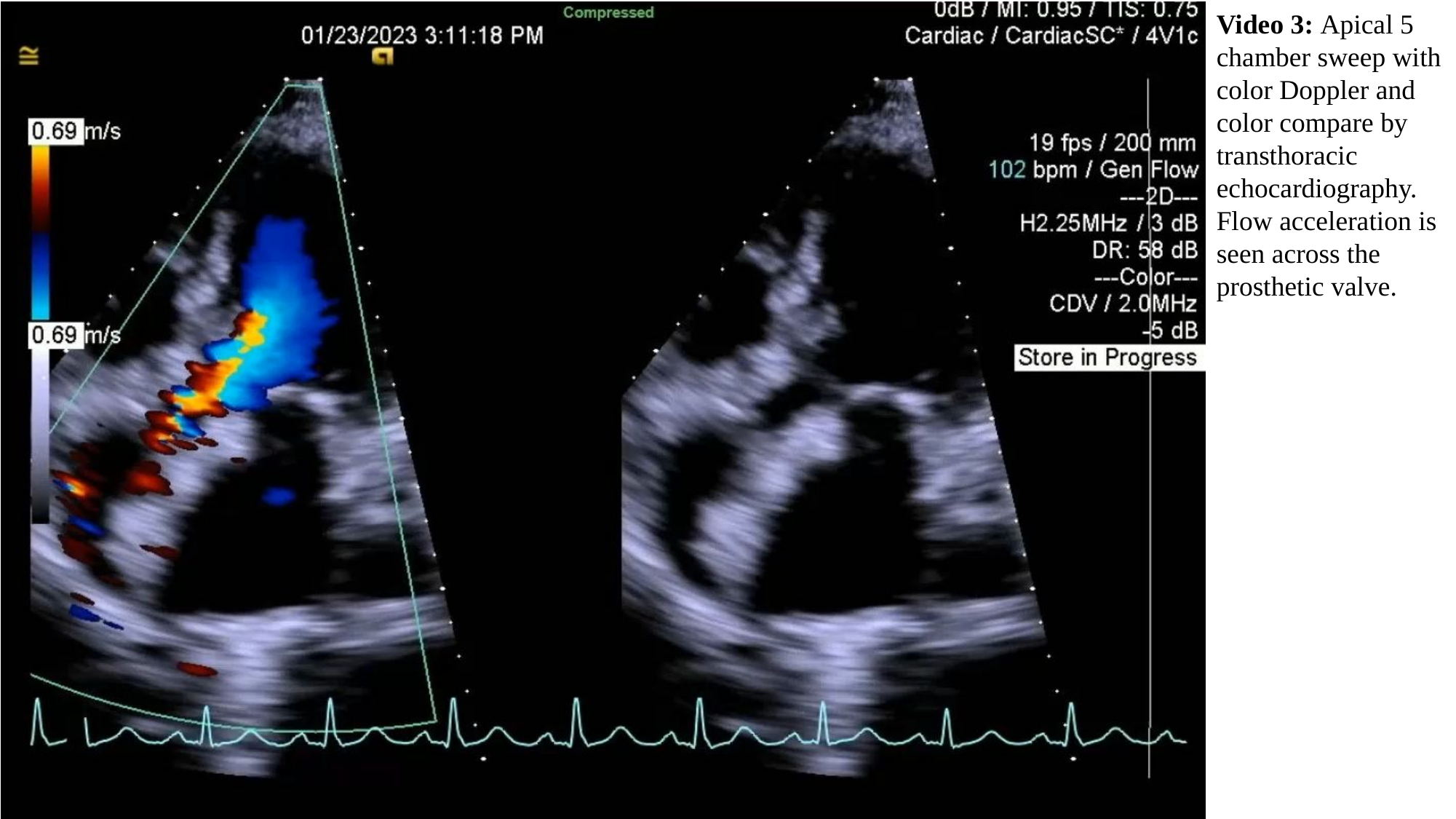

Video 3: Apical 5 chamber sweep with color Doppler and color compare by transthoracic echocardiography. Flow acceleration is seen across the prosthetic valve.
