# Supplementary material for: Differentiating Mobile Masses on Transcatheter Aortic Valve: Thrombi or Vegetations?
Source: Case Rep Cardiol. 2025 May 5;2025:9915565. doi: 10.1155/cric/9915565 (PMC12069840; doi:10.1155/cric/9915565)
Supplement: Supporting Information 4 — Video S4: Midesophageal, long-axis cine by transesophageal echocardiography. Mobile masses seen on the aortic aspect of the prosthetic valve. [file 9915565.f4.pptx]

## Slide 1
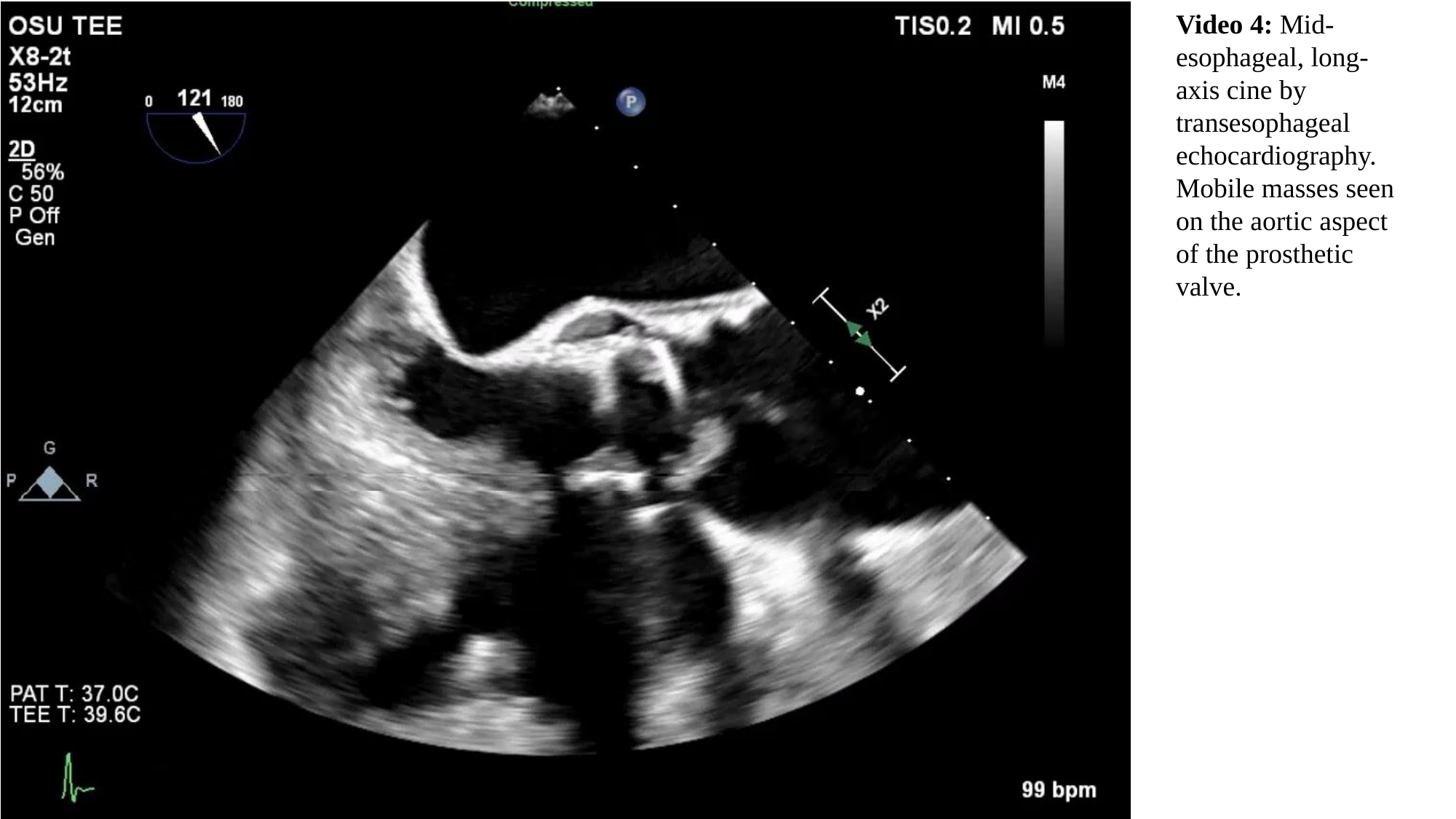

Video 4: Mid-esophageal, long-axis cine by transesophageal echocardiography. Mobile masses seen on the aortic aspect of the prosthetic valve.
