# Supplementary material for: Differentiating Mobile Masses on Transcatheter Aortic Valve: Thrombi or Vegetations?
Source: Case Rep Cardiol. 2025 May 5;2025:9915565. doi: 10.1155/cric/9915565 (PMC12069840; doi:10.1155/cric/9915565)
Supplement: Supporting Information 5 — Video S5: Midesophageal, long-axis cine with color Doppler by transesophageal echocardiography. Flow acceleration is seen across the prosthetic valve. [file 9915565.f5.pptx]

## Slide 1
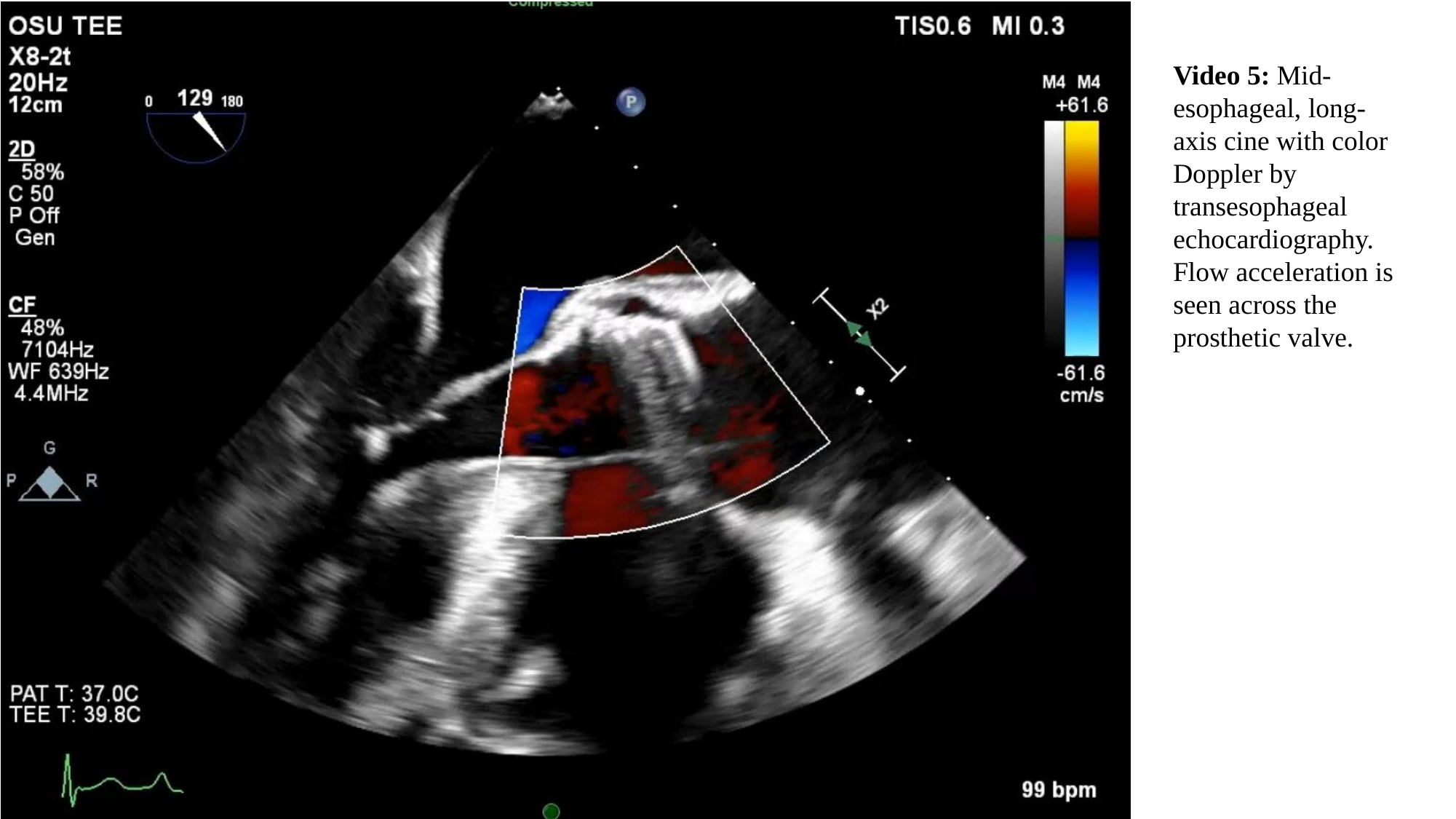

Video 5: Mid-esophageal, long-axis cine with color Doppler by transesophageal echocardiography. Flow acceleration is seen across the prosthetic valve.
